# Draft Genome Sequence of *Bacillus pumilus* Strain GM3FR, an Endophyte Isolated from Aerial Plant Tissues of *Festuca rubra* L.

**DOI:** 10.1128/genomeA.00085-17

**Published:** 2017-03-30

**Authors:** Jacqueline Hollensteiner, Anja Poehlein, Rolf Daniel, Heiko Liesegang, Stefan Vidal, Franziska Wemheuer

**Affiliations:** a Genomic and Applied Microbiology and Göttingen Genomics Laboratory, Institute of Microbiology and Genetics, Georg-August University Göttingen, Göttingen, Germany; b Agricultural Entomology, Department of Crop Sciences, Georg-August University Göttingen, Göttingen, Germany

## Abstract

Here, we report the draft genome sequence of *Bacillus pumilus* GM3FR, an endophytic bacterium isolated from aerial plant tissues of *Festuca rubra* L. The draft genome consists of 3.5 Mb and harbors 3,551 predicted protein-encoding genes. The genome provides insights into the biocontrol potential of *B. pumilus* GM3FR.

Plant-associated members of the genus *Bacillus* are well known for their plant growth–promoting functions (1, 2). Several *B. pumilus* strains are used as biocontrol agents against various phytopathogens (3–5). The genome of the endophytic *B. pumilus* strain GM3FR was sequenced to determine its potential as a biocontrol agent.

*Bacillus pumilus* GM3FR was isolated from surface-sterilized aerial tissues of healthy *Festuca rubra* L. plants. Genomic DNA of *B. pumilus* GM3FR was extracted using the MasterPure complete DNA purification kit (Epicentre, Madison, WI, USA). The obtained DNA was used to generate Illumina shotgun paired-end sequencing libraries. Sequencing was performed employing the MiSeq system and the MiSeq reagent kit version 3 (600 cycles) as recommended by the manufacturer (Illumina, San Diego, CA, USA). Quality filtering using Trimmomatic version 0.32 (6) resulted in 2,676,164 paired-end reads. *De novo* genome assembly was performed with the SPAdes genome assembler version 3.8.0 (7). The assembly resulted in 36 contigs (>500 bp) and an average coverage of 158-fold. The assembly was validated, and the read coverage was determined with QualiMap version 2.1 (8).

The draft genome of strain GM3FR consisted of 3,506,516 bp with an overall G+C content of 40.92%. Gene prediction and annotation were performed using Rapid Prokaryotic Genome Annotation (Prokka) (9). The draft genome harbored six rRNA genes, 68 tRNA genes, 1,897 protein-encoding genes with functional predictions, and 1,654 genes coding for hypothetical proteins. Multilocus sequence typing based on seven genes (*gyrB*, *rpoB*, *aroE*, *muL*, *pycA*, *pyrE*, and *trpB*) was performed according to Liu et al. (10): the analysis revealed that strain GM3FR belongs to the *B. pumilus* species group. The closest relative of GM3FR is *B. pumilus* SAFR-0.32, which has been isolated from an ultraclean spacecraft assembly facility (11).

A secondary metabolite gene prediction was performed using antiSMASH version 3.0.5 (12) and revealed nine potential gene clusters for secondary metabolite production. Six of these clusters showed no or weak (>40%) similarity to known clusters including genes encoding microcin, bacteriocin, terpene, siderophore-terpene, type I polyketide synthase (T1PKS), and a nonribosomal peptide synthetase (NRPS) T1PKS cluster. Moreover, a gene cluster was identified with 85% of the genes sharing similarity to a bacilysin gene cluster of *B. amyloliquefaciens* strain FZB42 (13). Bacilysin produced by strain FZB42 showed antimicrobial activities against the phytopathogens *Xanthomonas oryzae* (13) and *Erwinia amylovora* (14). An NRPS gene cluster was identified with 71% of genes sharing similarity to a lichenysin biosynthetic gene cluster identified in *B. licheniformis* DSM13, which encodes an antifungal substance (15). Finally, a head-to-tail bacteriocin gene cluster with 85% of the genes exhibiting similarities to a *skfA* gene cluster known from *B. subtilis* 168 (16) was detected. Thus, strain GM3FR contains multiple gene clusters assigned to secondary metabolism. Gene clusters affiliated to bacilysin and lichenysin have the potential to be biocontrol agents and to promote plant health. Moreover, genes involved in bacteriocin production could be beneficial for the control of other bacteria (17) and for plant growth (18).

## Accession number(s).

The whole-genome shotgun project has been deposited at DDBJ/ENA/GenBank under the accession number MKZN00000000. The version described here is the first version, MKZN01000000.
